# Comparison between bone–implant interfaces of microtopographically modified zirconia and titanium implants

**DOI:** 10.1038/s41598-023-38432-y

**Published:** 2023-07-10

**Authors:** Myint Kyaw Thu, Young Suk Kang, Jeong Min Kwak, Ye-Hyeon Jo, Jung-Suk Han, In-Sung Luke Yeo

**Affiliations:** 1 Department of Prosthodontics, School of Dentistry and Dental Research Institute, Seoul National University, 101, Daehak-ro, Jongro-gu, Seoul, 03080 Korea; 2 618th Medical Company (Dental Area Support)/Dental Health Activity-Korea, Camp Humphreys, APO, AP 96297 USA; 3 Dental Research Institute, Seoul National University, Seoul, 03080 Korea

**Keywords:** Implants, Dental biomaterials, Dental implants

## Abstract

The aim of this study was to investigate the surface characteristics and evaluate the bone–implant interfaces of injection molded zirconia implants with or without surface treatment and compare them with those of conventional titanium implants. Four different zirconia and titanium implant groups (n = 14 for each group) were prepared: injection-molded zirconia implants without surface treatment (IM ZrO_2_); injection-molded zirconia implants with surface treatment via sandblasting (IM ZrO_2_-S); turned titanium implants (Ti-turned); and titanium implants with surface treatments via sandblasting with large-grit particles and acid-etching (Ti-SLA). Scanning electron microscopy, confocal laser scanning microscopy, and energy dispersive spectroscopy were used to assess the surface characteristics of the implant specimens. Eight rabbits were used, and four implants from each group were placed into the tibiae of each rabbit. Bone-to-implant contact (BIC) and bone area (BA) were measured to evaluate the bone response after 10-day and 28-day healing periods. One-way analysis of variance with Tukey’s pairwise comparison was used to find any significant differences. The significance level was set at α = 0.05. Surface physical analysis showed that Ti-SLA had the highest surface roughness, followed by IM ZrO_2_-S, IM ZrO_2_, and Ti-turned. There were no statistically significant differences (*p* > 0.05) in BIC and BA among the different groups according to the histomorphometric analysis. This study suggests that injection-molded zirconia implants are reliable and predictable alternatives to titanium implants for future clinical applications.

## Introduction

Implant dentistry has become a predictable and reliable treatment modality for restoring the esthetics and functions of partially and completely edentulous patients. For successful outcomes of implant-supported dental prostheses, osseointegration, the biological fixation of an implant defined as direct bone-to-implant contact (BIC) without an intervening connective tissue layer, must be achieved^1,2^. Therefore, BIC is a critical indicator for successful osseointegration, which governs the long-term clinical success and survival of implant-supported dental prostheses^3^. For the optimal BIC, the surface characteristics, both physical and chemical, of dental implants play a critical role in the osseointegration process.

Over the last 40 years, both commercially pure titanium (Ti) and titanium alloys have been used for dental implants due to their excellent biocompatibility, mechanical strength, chemical inertness, and high clinical success rates^4^. Since the 1990s, various microtopographical modification methods for Ti implant surfaces have been introduced by implant manufacturers to accelerate early osseous integration and increase the resistance of the bone–implant interface to functional loading^5^. Among them, roughening Ti implant surfaces at the microscopic level with sandblasting and acid-etching has become very popular due to its high success rate^6^. However, the main disadvantage of Ti implants is their dark grayish color, which may lead to esthetic compromise if buccal bone loss and unfavorable soft-tissue response in gingival biotypes occur^7^.

Therefore, researchers have developed zirconia (zirconium dioxide, ZrO_2_) implants in the last 2 decades to mitigate the esthetic disadvantage of titanium implants^8,9^. The main advantage of ZrO_2_ implants is their tooth-like color, which makes them superior to Ti implants in esthetically critical locations, such as the maxillary anterior area. In addition, they possess promising characteristics, such as a high resistance to wear and corrosion, high fracture resistance and flexural strength, biocompatibility, minimal ion-release, and reduced bacterial adhesion and plaque accumulation^10–12^. However, ZrO_2_ also has disadvantages, such as a sensitivity to low-temperature degradation (aging), vulnerability to subcritical bending and crack growth, and lower fracture resistance, compared to titanium^13,14^. It is also challenging to roughen ZrO_2_ implant surfaces because of their hardness^15^. In the literature, various attempts to modify ZrO_2_ surfaces have been reported, such as sandblasting, etching with hydrochloric or hydrofluoric acids^16^, the aggregation of bioactive materials such as hydroxyapatite^17^, plasma spraying^18^, ultraviolet radiation to induce the hydrophilicity of ZrO_2_^19^, and selective infiltration-etching techniques to create a nanoporous surface^20^.

Although both the additive manufacturing and subtractive (milling) manufacturing of ZrO_2_ by computer-aided design and computer-aided manufacturing (CAD/CAM) technology have been studied frequently, as reported in the literature^21–23^, the injection molding of ZrO_2_ has been reported in the literature since the 1980s^24^. This technique is a type of plasticity-shaping technique that utilizes a binder, plasticizer, lubricant, and coupling agent^25^. The process begins with compounding fine ceramic powders with a blend of polymers or wax in solvent. Then, the organic binders embedded in the molds are removed via thermal pyrolysis or solvent detracting before sintering^26^. Injection molding is suitable for making relatively fine ceramic parts because it offers dimensional reproducibility, requires little or no modification, and produces parts with a sufficient quality for clinical applications^27^. In particular, this technique allows the mass production of ceramic parts at low cost and near-net-shape formation^28^.

Previous animal studies and various case reports have indicated that the osseointegration of ZrO_2_ implants is similar to or even superior to that of Ti implants^29–31^. However, the effects of different surface characteristics (i.e., smooth vs. rough) of ZrO_2_ implants on the osseointegration process are still largely unknown^32^. Furthermore, there is no report on the early bone response around injection-molded ZrO_2_ implants compared to that around Ti implants. This in vivo study aimed to compare the bone–implant interfaces of injection-molded ZrO_2_ implants and computer numerical control (CNC)-machined Ti implants. Two types of surfaces were prepared for each material: sandblasted and nonsandblasted surfaces for the injection-molded ZrO_2_ implants and sandblasted, large-grit, acid-etched (SLA) and non-SLA surfaces for the CNC-machined Ti implants. The surface characteristics of the implants were also investigated. The hypothesis underlying this study was that the hard-tissue response to the injection-molded ZrO_2_ implants is similar to the osseointegration of Ti implants with SLA surfaces, which are used in dental clinics worldwide.

## Methods

### Preparation of implant samples

A total of 56 screw-shaped dental implant samples (28 ZrO_2_ implants (one-piece) and 28 Ti implants) of the same macroscopic shape and dimensions (a diameter of 3.4 mm and a length of 8 mm) were used in this study. Also, ten ZrO_2_ discs, which were 15 mm in diameter and 1 mm in thickness, were prepared to find some phase transition of ZrO_2_ after surface modification. The ZrO_2_ implants were manufactured using an injection molding technique (Vatech Acucera, Seoul, Korea). This process involves mixing zirconia powders with modifiers and shaping a uniform and homogeneous mixture into a mold. The Ti implants were manufactured by a computer numerical controlled (CNC) milling technique (Deep Implant System, Inc., Seongnam, Korea). Disc-shaped green compacts were prepared by cold isostatic press of powder mixtures and then sintered. Surface modification with sandblasting was performed on half of the ZrO_2_ implants and the ZrO_2_ discs, while half of the Ti implant samples were surface-modified with SLA treatment, i.e., sandblasted with large-grit alumina (Al_2_O_3_) particles and etched with hydrochloric acid (SLA surface; Deep Implant System, Inc., Seongnam, Korea). After sandblasting, the ZrO_2_ implants (14 implants) and discs (5 discs) were treated by hot isostatic pressing (HIP) at 1380 °C and 138 MPa. Then, the samples were divided into four experimental groups:

1. Group 1 = IM ZrO_2_ (injection-molded ZrO_2_ implants or ZrO_2_ discs without sandblasting modification)
2. Group 2 = IM ZrO_2_-S (injection-molded ZrO_2_ implants or ZrO_2_ discs with sandblasting modification)
3. Group 3 = Ti-turned (Ti implants without SLA modification as a negative control)
4. Group 4 = Ti-SLA (Ti implants with SLA modification as a positive control)

### Assessment of surface characteristics

The implant sample surfaces were photographed by field emission-scanning electron microscopy (FE-SEM; S-4700, Hitachi, Tokyo, Japan). Surface parameters for the sample topography were measured by confocal laser scanning microscopy (CLSM; LSM 800, Carl Zeiss AG, Oberkochen, Germany). The acquired images were analyzed using ConfoMap software, and specific areas of interest were selected. Subsequently, the surface topography was quantified in terms of Sa (arithmetical mean height of a surface), the absolute value of the difference in height of each point compared to the arithmetical mean of the surface, and Sdr (developed interfacial area ratio), the proportion of the additional surface area contributed by the texture within the defined planar area. Each sample was analyzed at 3 selected sites (the upper, middle and lower flank), the values of which were averaged, and the average value was assigned as the representative value for the sample^22,33^. The topography was measured in terms of Sa values (arithmetical mean height) and Sdr values (developed interfacial area ratio). In addition, the chemical composition of each sample was analyzed with an energy-dispersive spectroscopy (EDS) device (EMAX, Horiba, High Wycombe, United Kingdom). For evaluating the phase transition of ZrO_2_, the ZrO_2_ disc surfaces were analyzed using a high resolution X-ray diffractometer (SmartLab, Rigaku, Tokyo, Japan) with Cu Kα radiation (wavelength = 1.54 Å) and 45 kV/200 mA. To quantify the molar fraction of the content of monoclinic ZrO_2_ ($X_{m}$), the following equations were employed:

$$X_{m} = \frac{{I_{m} \left( {111} \right) + I_{m} \left( {\overline{1}11} \right)}}{{I_{m} \left( {111} \right) + I_{m} \left( {\overline{1}11} \right) + I_{t} \left( {101} \right)}}$$

where $I_{t}$ and $I_{m}$ represent the intensity of the tetragonal $\left( {101} \right)$ and monoclinic $\left( {111} \right)$ and $\left( {\overline{1}11} \right)$ peaks^34,35^. The peak intensity was obtained using MDI Jade 6 software (Materials Data Inc., Livermore, CA, USA).

### In vivo surgery

**Figure 1:**
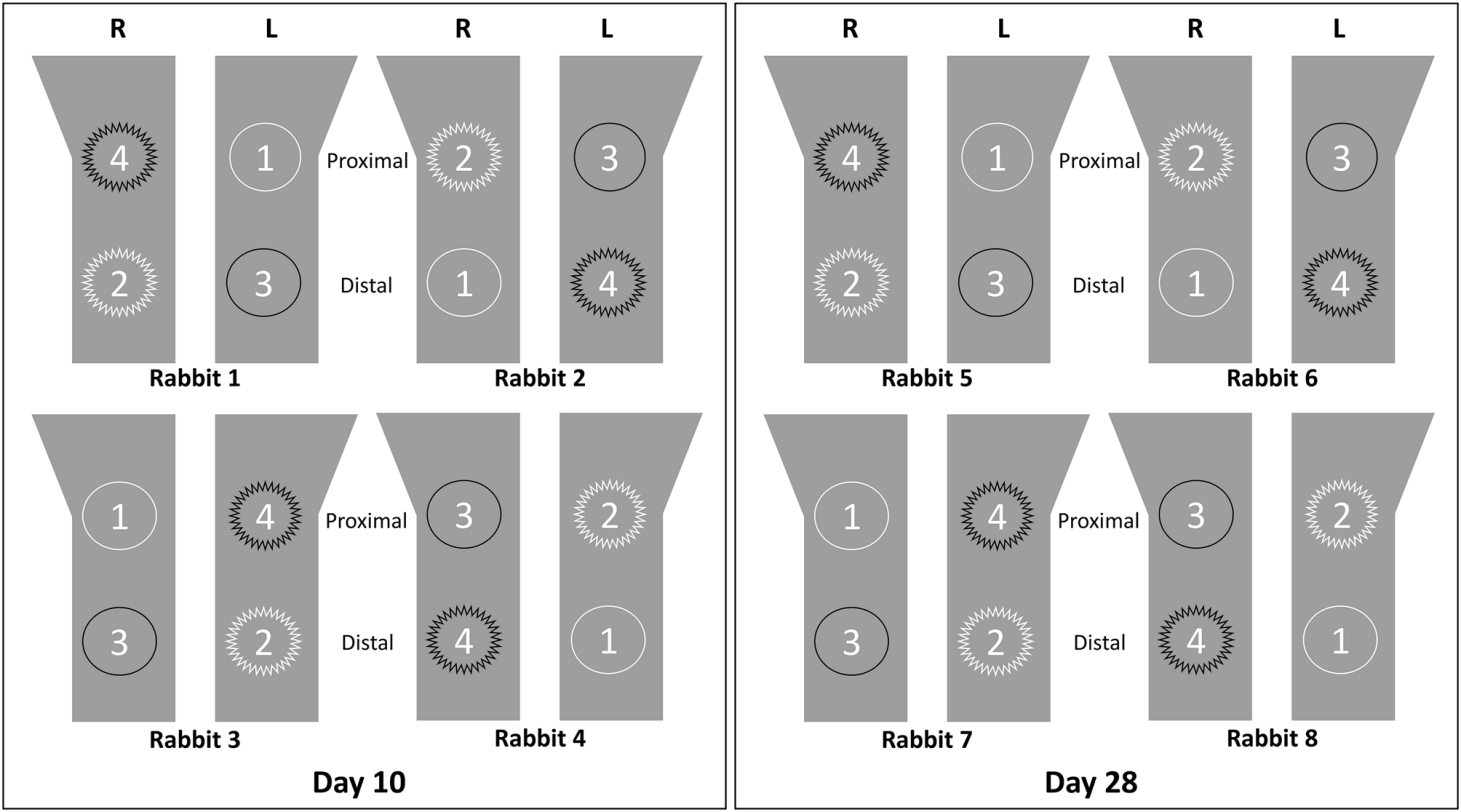
Four implant groups used in this study. 1: injection-molded ZrO_2_ implant; 2: sandblasted injection-molded ZrO_2_ implant; 3: turned Ti implant; 4: SLA Ti implant. This schematic diagram shows that these implants were placed in rabbit tibiae according to the split-plot design. The saw-edged circles represent the implants whose surfaces were microtopographically modified, while the smooth edged ones represent implants with no microtopographical modification. Black represents Ti, and white represents ZrO_2_. R = right tibia; L = left tibia.

Eight male New Zealand white rabbits (age: 3–4 months old; weight: 2.5–3.0 kg) were used in this in vivo study, which was approved by the Institutional Animal Research Ethics Committee of Cronex (CRONEX-IACUC: 202108011, Hwaseong, Korea) and conducted according to the Animal Research: Reporting In Vivo Experiments (ARRIVE) guidelines^36^. All methods were performed in accordance with the relevant guidelines and regulations. The experimental animals acclimatized in separate cages for two weeks before surgery. Each rabbit received four implant samples; two implants were placed in each tibia bone of the hind legs. The ZrO_2_ and Ti implants were inserted based on the split-plot design (Fig. 1). For anesthesia, a combination of 15 mg/kg tiletamine hydrochloride and zolazepam hydrochloride (Zoletil 50; Virbac Korea Co., Ltd., Seoul, Korea) and 5 mg/kg xylazine (Rompun; Bayer Korea, Ltd., Seoul, Korea) was administered intramuscularly. Then, the hind legs were shaved and disinfected with an antiseptic surgical scrub of 7.5% povidone-iodine (Betadine; Korea Pharma, Seoul, Korea). After site preparation, local infiltration anesthesia of 2% lidocaine hydrochloride with 1:100,000 epinephrine (Yuhan Company, Seoul, Korea) was administered at the surgical sites. The tibial bones were exposed by full-thickness incisions from the skin to the periosteum. The surgical sites on the tibiae bones were prepared with rotating implant drills and engines under copious irrigation with sterile saline solution. The final drill size was 3.0 mm, and 32 sample implants from experimental groups 1, 2, 3 and 4 were placed with primary stability (≥ 20 Ncm) using a torque wrench, according to the manufacturer’s instructions. After the implant placement surgeries, the muscle and fascia were sutured with resorbable 4–0 Vicryl sutures (Coated Vicryl; Ethicon, Raritan, NJ, United States), and the outer dermis was closed with nylon (Blue nylon; Ailee, Busan, Korea). All rabbit specimens were housed in individual cages and administered the postoperative antibiotic prophylaxis of enrofloxacin (Biotril, Komipharm International, Siheung, Korea).

### Assessment of histology and histomorphometry

Four rabbits were sacrificed 10 days after the implant placement (Rabbit 1–4), and the remaining 4 rabbits were sacrificed 28 days after the implant placement (Rabbit 5–8) by an overdose of potassium chloride intravenously under anesthesia for histologic assessment. The bones and connective tissues that surrounded the implant samples were surgically harvested en bloc. These blocks were fixed in 10% neutral buffered formalin for two weeks and then dehydrated with ethanol, followed by embedding in light-curing resin (Technovit 7200 VLC, Kulzer, Wehrheim, Germany). A series of cutting and grinding devices (EXAKT system; EXAKT Apparatebau, Norderstedt, Germany) was used to cut and grind the embedded blocks into slides with a thickness of less than approximately 50 μm^22,37^. The slides were stained with modified Goldner’s Masson trichrome staining solution for examination under a light microscope. This staining technique allows for easy discrimination between newly formed bone (stained red) and exiting mature bone (stained blue)^38^. The bone-to-implant interfaces were measured by the degree of BIC and bone area (BA) at the best three consecutive threads^22,39^. The histologic evaluation was performed using a light microscope (DM2700M, Leica Microsystems CMS GmbH, Wetzlar, Germany) and an attached digital camera (DMC5400, Leica Microsystems CMS GmbH, Wetzlar, Germany). An image analysis system (ImageJ 1.60, NIH, Bethesda, MD, United States) was used to analyze the acquired images.

### Statistical analysis

Most of the outcome variables for data normalization were accepted when the Shapiro‒Wilk test was used (*p* > 0.05). Descriptive statistics are shown as the means and standard deviations (SDs). One-way analysis of variance (ANOVA) was used to analyze differences in the mean values of the surface parameters and histomorphometric data of the different types of implants in this study. When a significant difference was found, Tukey's honestly significant difference (HSD) was further applied for a pairwise comparison. In addition, the independent t test was used to compare the BIC and BA values of the 2 healing periods (10 days vs. 28 days). Statistical software, R version 4.1.0 (R Foundation for Statistical Computing, Vienna, Austria), was used for all statistical evaluations. The statistical significance level was set at α = 0.05.

### Ethics approval and consent to participate

Ethical approval was sought from the Institutional Animal Care and Use Committee of CRONEX Co., Ltd., Hwaseong, Korea, and the animal experiment was conducted in accordance with the Animal Reporting in Vivo Experiments (ARRIVE) guidelines (202108011).

### Consent for publication

All authors are aware of the publication of this work.

## Results

### Surface characteristics

**Figure 2:**
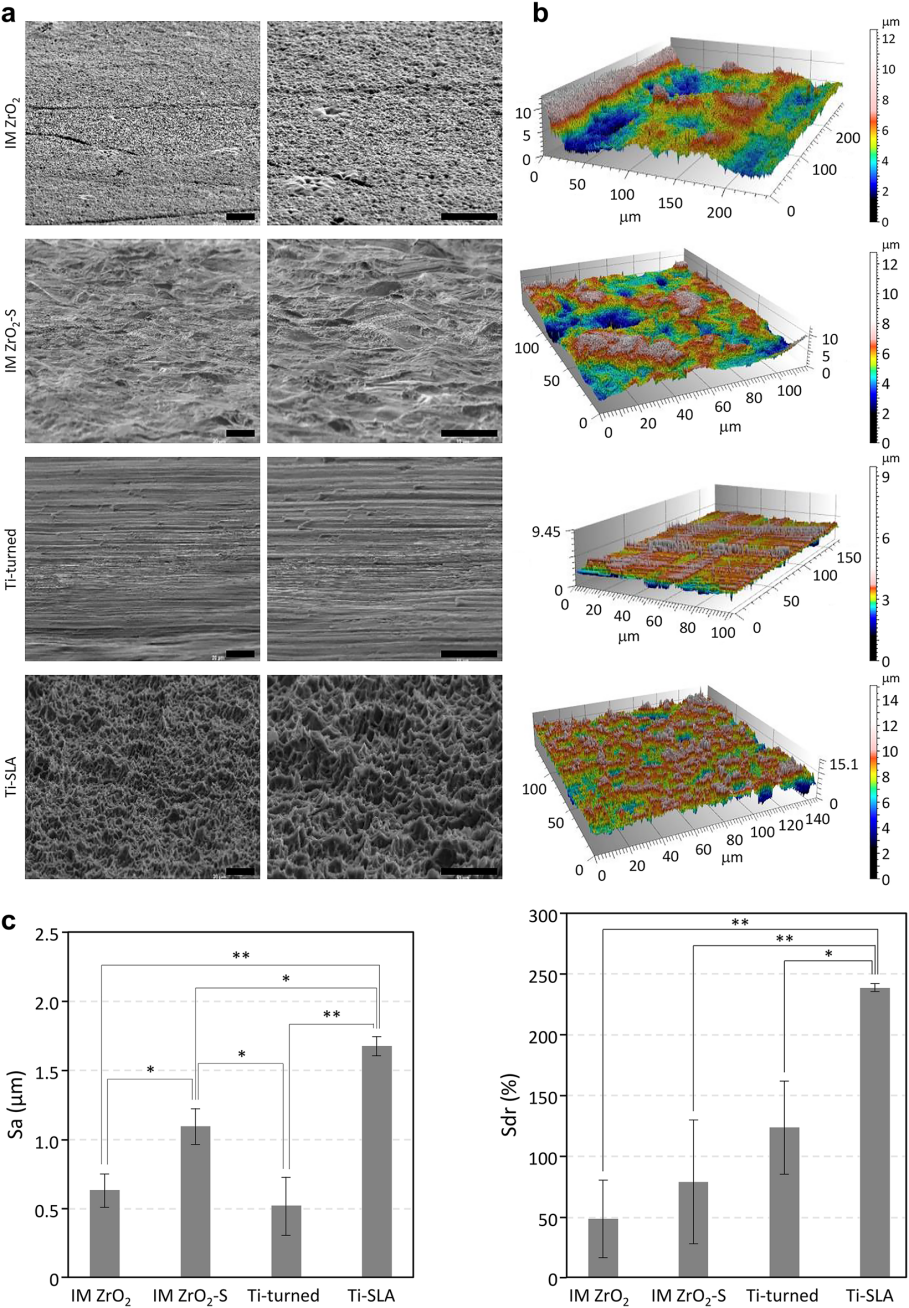
Surface characteristics of the ZrO_2_ and Ti specimens investigated in this study. (**a**) FE-SEM images of the surfaces of IM ZrO_2_ (injection-molded zirconia without surface treatment), IM ZrO_2_-S (injection-molded zirconia implant with sandblasting surface treatment), Ti-turned (turned titanium implant), and Ti-SLA (SLA titanium implant) at 2000× (left) and 5000× (right) magnifications. Scale bars = 10 μm. (**b**) CLSM images of surface roughness of implant surfaces (3D views of color-coded height maps with texture overlay). (**c**) Surface roughness parameters (Sa and Sdr) of the implant surfaces. Data are presented as the means ± SDs (n = 3). *p < 0.05 and **p < 0.01.

The FE-SEM images of the implant surfaces of the 4 different experimental groups are shown in Fig. 2A. The IM ZrO_2_ (Group 1) implants showed microcracks, porosities, and grain structures that are typically observed for sintered ZrO_2_. In contrast, the IM ZrO_2_-S (Group 2) implants showed very rough surfaces with slate-like profiles and lower porosities. The Ti-turned (Group 3: negative control) implants showed smooth and flat surfaces with continuous straight lines that ran in one direction. However, the Ti-SLA (Group 4: positive control) implants exhibited a rough, irregular, porous, and honeycomb-like appearance.

The surface roughness parameters of the experimental groups were measured in terms of Sa and Sdr values (Fig. 2B,C). Based on the mean Sa values and SDs, the Ti-SLA implants had the highest Sa value (1.68 μm ± 0.07 μm), followed by the IM ZrO_2_-S implants (1.10 μm ± 0.13 μm), IM ZrO_2_ implants (0.64 μm ± 0.12 μm), and Ti-turned implants (0.52 μm ± 0.21 μm). There were statistically significant differences among the Sa values of all groups, except between the IM ZrO_2_ and Ti-turned groups. The mean Sdr values of the Ti-SLA implants (238.78% ± 3.03%) were significantly higher than those of the other three groups. However, there were no significant differences among the Sdr values of these three groups: IM ZrO_2_ (49.04% ± 31.68%), IM ZrO_2_-S (78.77% ± 50.02%), and Ti-turned (123.66% ± 37.54%).

**Figure 3:**
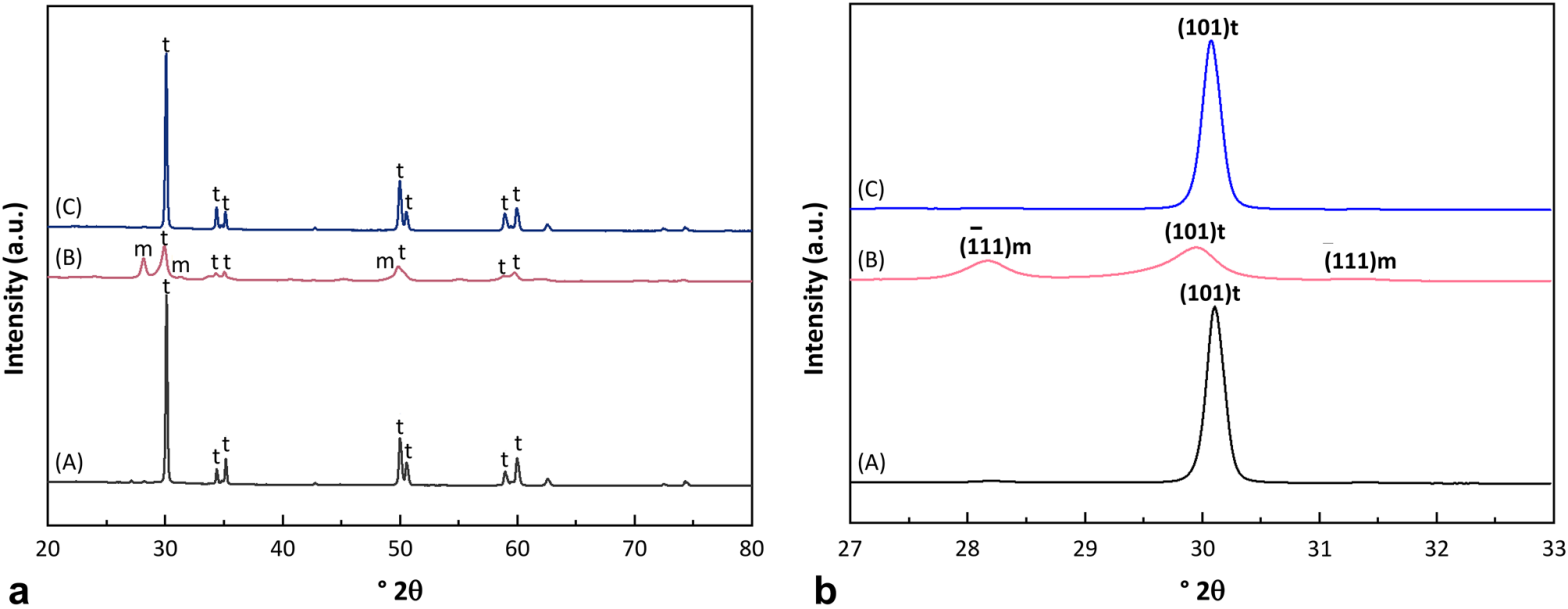
X-ray diffraction (XRD) patterns of the ZrO_2_ discs (**a**) in the range of 20–80° 2θ are presented. More detailed XRD patterns are also shown in the range of 27–33° 2θ (**b**). Compared to the XRD peaks of the ZrO_2_ discs before sandblasting (A), the presence of the monoclinic phase was detected after the ZrO_2_ discs were sandblasted (B). The monoclinic phase disappeared upon hot isostatic pressing of the sandblasted ZrO_2_ discs (C).

The EDS findings are shown in Table 1. Titanium (Ti), carbon (C), and oxygen (O) were detected in the Ti implants, while zirconium (Zr), carbon (C), and oxygen (O) were detected in the ZrO_2_ implants. The X-ray diffraction patterns of the ZrO_2_ discs are shown in Fig. 3. ZrO_2_ discs without sandblasting modification only presented the tetragonal ZrO_2_ peak. ZrO_2_ discs with sandblasting modification presented the $\left( {111} \right)$ and $\left( {\overline{1}11} \right)$ peaks for the monoclinic phase (m-ZrO_2_) and the $\left( {101} \right)$ peak for the tetragonal phase (t-ZrO_2_). However, it was clearly seen that while the ZrO_2_ discs with sandblasting modification presented both the peaks corresponding to the tetragonal and monoclinic phases, only the peak of tetragonal phase was detected after HIP. As seen in Table 2, the rate of the tetragonal-to-monoclinic phase transformation increased with sandblasting modification. It was found that the amount of m-ZrO_2_ was 40.32% of the total ZrO_2_ after sandblasting modification. After HIP, this amount of monoclinic phase was totally transformed into t-ZrO_2_.

**Table 1:** Element composition analysis of the ZrO_2_ and Ti implant surfaces according to energy dispersive spectroscopy.

Group (n = 3)	Atomic percentage (%)				
	C	O	N	Zr	Ti
IM ZrO_2_	56.67	07.47	0.00	35.85	00.00
IM ZrO_2_-S	48.58	24.56	0.00	26.85	00.00
Ti-turned	05.56	11.86	0.00	00.00	82.54
Ti-SLA	04.04	14.24	0.00	00.00	81.72

*C* carbon, *O* oxygen, *N* nitrogen, *Zr* zirconium, *Ti* titanium.

**Table 2:** The tetragonal-to-monoclinic phase transformation in ZrO_2_ discs with and without sandblasting modification and after HIP.

m-ZrO_2_*	Without sandblasting	With sandblasting	After HIP^†^
Mean (SD)	1.03 (0.09)	40.32 (5.97)	0.50 (0.07)

*Monoclinic ZrO_2_.

^†^Hot isostatic pressing.

### Analysis of histology and histomorphometry

**Figure 4:**
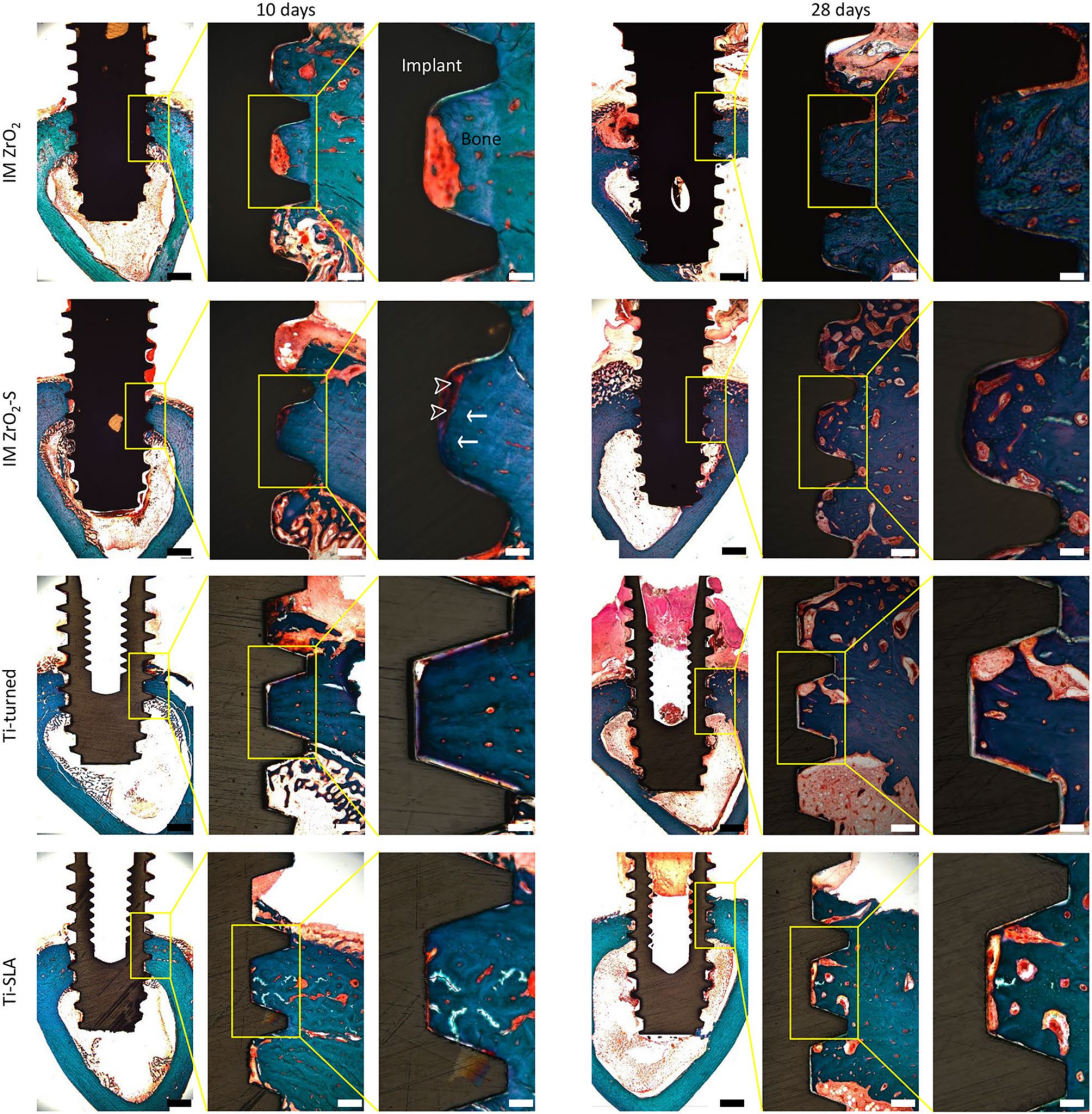
Histological views and histomorphometric data for bone responses to the ZrO_2_ and Ti implants. Light microscopic views of the implants with modified Goldner’s Masson trichrome staining at 10 and 28 days after installation into rabbit tibiae (scale bars from left to right: 1 mm at × 12.5, 200 μm at × 50, and 100 μm at × 100 magnification). The exiting mature bone was stained blue (white arrow), and new immature bone or osteoid was stained red (white-edged arrowhead) at the bone–implant interfaces.

All the implant samples successfully osseointegrated after 10 days of healing and 28 days of healing (Supplementary Fig. S1). In the histological analysis of the ZrO_2_ and Ti implants stained with modified Goldner’s Masson trichrome staining solution, new bone formation was found along the bone–implant interfaces. In the cortical bone area of the tibiae, the exiting mature bones were stained blue, while newly formed immature bones were stained red and detected at the implant threads and around the mature bone (Fig. 4).

**Figure 5:**
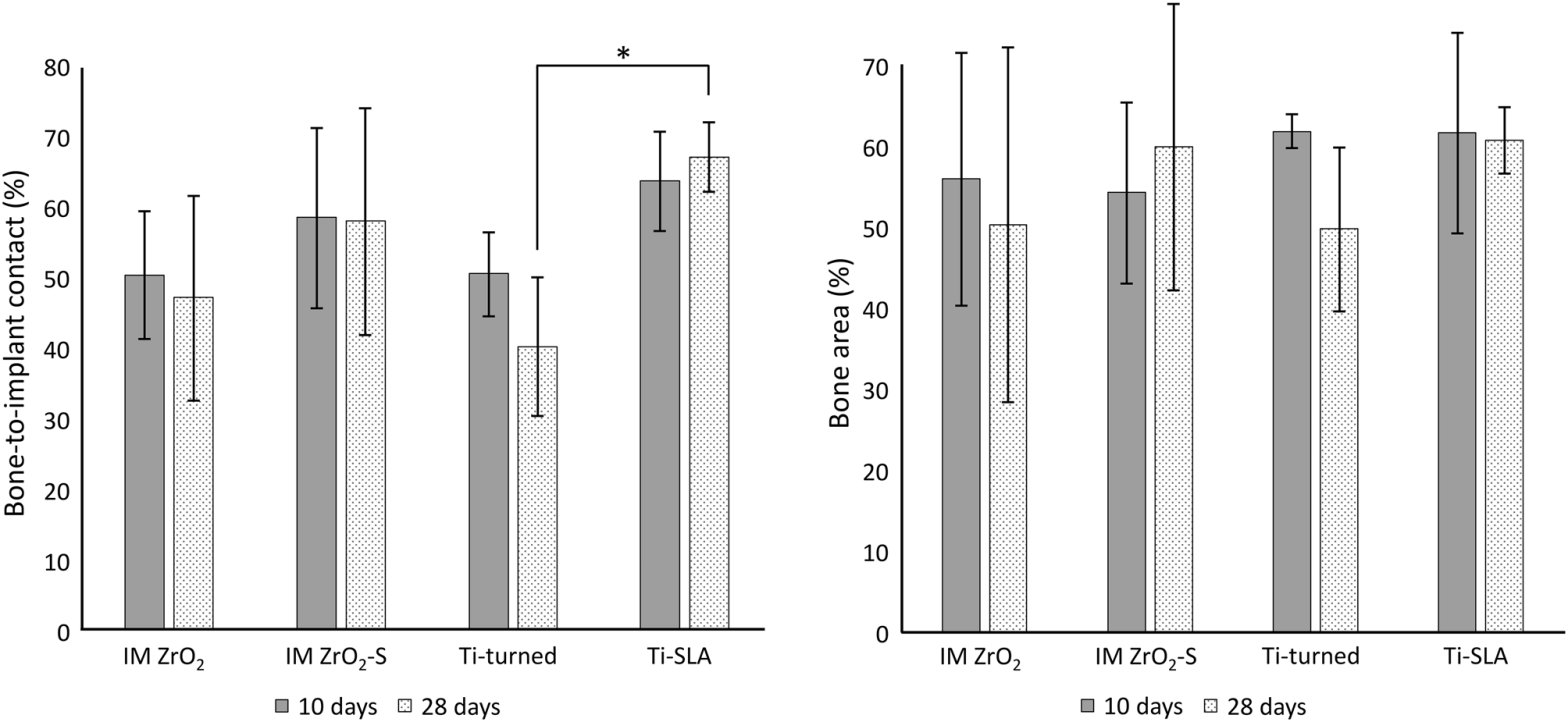
The mean and standard deviation of bone-to-implant contact (left) and bone area (right) ratios of the implants at 10 and 28 days after installation (n = 4). *p < 0.05.

The mean BIC and BA values and SDs of the ZrO_2_ and Ti implants at the 10-day and 28-day marks are shown in Fig. 5 (Supplementary Tables S1, S2). Although the BICs (%) of the IM ZrO_2_-S and Ti-SLA implants were higher than those of the Ti-turned and IM ZrO_2_ implants at the 10-day mark, the differences were not statistically significant. At the 28-day mark, the BIC (%) of the Ti-SLA implants was significantly higher than that of the Ti-turned implants (*p* = 0.04). However, the other groups showed no significant differences. When the mean values and SDs of BA (%) were calculated, no statistically significant differences were found among the ZrO_2_ and Ti implants for both healing periods (*p* > 0.05) (Fig. 5).

## Discussion

The roughness parameters (Sa and Sdr) of the surface-treated implants (Groups 2 and 4) were significantly higher than those of the ZrO_2_ and Ti implant groups without surface treatment (Groups 1 and 3). The authors also found that the BIC% and BA% of the IM ZrO_2_ implants were not significantly different from those of the Ti implants (both Ti-turned and Ti-SLA) after 10-day and 28-day healing periods. These results are in agreement with the findings of previous studies that compared SLA-treated IM ZrO_2_ and Ti implants in mini pig maxillae models^40^, canine models^41^, and rabbit tibia models^42^. Another significant finding was that the surface-treated ZrO_2_ implants showed enhanced bone integration at the implant surface compared to the untreated ZrO_2_ implants. This finding is in line with the results of other studies by Mihatovic in 2017 and Schünemann in 2019^43,44^. In addition, the Ti-SLA implants demonstrated a significantly higher bone response than the Ti-turned implants. This finding is also comparable to findings in other studies^45,46^. It can be deduced that the rough surface microtopography of ZrO_2_ implants generated by the proper surface modification can influence early bone response and long-term sustainability.

In this study, the authors used 10-day and 28-day healing periods for bone formation in a rabbit tibia model based on previous studies^47,48^. These healing periods of rabbit specimens are equivalent to 1-month and 3-month healing periods of humans, according to Roberts, who demonstrated that the bone healing of rabbits was approximately three times faster than that of humans^49^. Although rabbits are regarded as commonly used and well-established animal models for investigating the osseointegration process, some disadvantages include site limitations and mismatched microstructures when compared to those of human bone. In addition, histomorphometric analysis has some disadvantages, although BIC and BA have become the most popular parameters. For example, they are only measured in 2 dimensions without consideration of the entire implant, and they may be affected by the quality and quantity of the surrounding bones^50^. There were slight discrepancies in the geometry of the ZrO_2_ and Ti implants that might have affected the tissue reaction at the bone–implant interface. The small sample size employed in this study also presents a limitation. The absence of significant differences observed in the in vivo experiments could be attributed to this small sample size. Further studies are needed to develop methods for the predictable early bone response and long-term osseointegration of IM ZrO_2_ implants with a double-blinded study design and a larger sample size. Furthermore, it is needed to investigate the optimal surface roughness of ZrO_2_ implants for osseointegration in subsequent studies.

## Conclusion

Based on the findings of this study, it is evident that appropriate surface treatment of ZrO_2_ implants is essential for promoting early peri-implant bone formation. Considering the recognized advantages of simplicity, mass production capability, and economic feasibility associated with the injection molding technique, utilizing injection molded zirconia dental implants, coupled with suitable surface modifications, could present a promising alternative to conventionally manufactured titanium dental implants for future clinical applications.

## Supplementary Information

Supplementary Information.

## Data Availability

All data generated or analyzed during this study are included in this published article.

## Abbreviations

Ti: Titanium
ZrO_2_: Zirconia; zirconium dioxide
SLA: Sandblasted with large-grit particles and acid-etched
IM ZrO_2_: Injection-molded zirconia implant
IM ZrO_2_-S: Injection-molded zirconia implant with sandblasting surface treatment
Ti-turned: Turned titanium implant
Ti-SLA: Titanium implant with SLA surface treatment
BIC: Bone-to-implant contact
BA: Bone area
CNC: Computer numerical control
FE-SEM: Field emission-scanning electron microscopy
CLSM: Confocal laser scanning microscopy
EDS: Energy dispersive spectroscopy
ARRIVE: Animal research: reporting in vivo experiments
XRD: X-ray diffraction
m-ZrO_2_: Monoclinic zirconia
t-ZrO_2_: Tetragonal zirconia
