# Supplementary material for: Comparison between bone–implant interfaces of microtopographically modified zirconia and titanium implants
Source: Sci Rep. 2023 Jul 10;13:11142. doi: 10.1038/s41598-023-38432-y (PMC10333275; doi:10.1038/s41598-023-38432-y)
Supplement: Supplementary file 1 — Supplementary Information. [file 41598_2023_38432_MOESM1_ESM.docx]

**Comparison between bone-implant interfaces of microtopographically modified zirconia and titanium implants**

Myint Kyaw Thu^1^, Young Suk Kang^2^, Jeong Min Kwak^1^, Ye-Hyeon Jo^3^, Jung-Suk Han^1^, In-Sung Luke Yeo^1,*^

^1^Department of Prosthodontics, School of Dentistry and Dental Research Institute, Seoul National University, Seoul 03080, Korea

^2^618^th^ Medical Company (Dental Area Support)/Dental Health Activity-Korea, Camp Humphreys, APO, AP 96297, USA

^3^Dental Research Institute, Seoul National University, Seoul 03080, Korea

Myint Kyaw Thu, Young Suk Kang and Jeong Min Kwak contributed equally to this work.

^*^**Corresponding Author**

In-Sung Luke Yeo, DDS, MSD, PhD, Professor

Department of Prosthodontics, School of Dentistry and Dental Research Institute, Seoul National University, 101, Daehak-ro, Jongro-gu, Seoul 03080, Korea

Tel: +82-2-2072-2661, Fax: +82-2-2072-3860, Email: pros53@snu.ac.kr

**Supplementary figures**

IM ZrO_2_

IM ZrO_2_-S

Ti-turned

Ti-SLA

Rabbit 1

Rabbit 2

Rabbit 3

Rabbit 4

**10 Days**

**a**

IM ZrO_2_

IM ZrO_2_-S

Ti-turned

Ti-SLA

Rabbit 5

Rabbit 6

Rabbit 7

Rabbit 8

**28 Days**

**b**

**Figure S1.** Histological images showing bone responses to the ZrO_2_ and Ti implants from all animals. (**a**) Light microscopic views of the implants stained with modified Goldner’s Masson trichrome at 10 days after installation into rabbit tibiae (from left to right: Rabbit 1–4). (**b**) Light microscopic views of the implants stained with modified Goldner’s Masson trichrome at 28 days after installation into rabbit tibiae (from left to right: Rabbit 5–8). Scale bars: 200 μm.

**Supplementary tables**

**Table S1.** Raw data of bone-to-implant contact and bone area ratios at 10 days after bone healing

| Rabbit No. | BIC (%) | | | | | BA (%) | | | |
| --- | --- | --- | --- | --- | --- | --- | --- | --- | --- |
|  | ZrO_2_* | ZrO_2_-S^†^ | Ti-T^‡^ | Ti-SLA | ZrO_2_ | | ZrO_2_-S | Ti-T | Ti-SLA |
| 1 | 47.25 | 67.30 | 51.46 | 65.01 | 37.09 | | 58.37 | 59.34 | 51.94 |
| 2 | 57.58 | 53.67 | 45.19 | 70.80 | 70.18 | | 44.32 | 62.77 | 79.23 |
| 3 | 38.63 | 70.41 | 46.83 | 53.88 | 49.28 | | 68.33 | 64.22 | 61.60 |
| 4 | 57.65 | 42.65 | 58.62 | 64.70 | 67.25 | | 46.49 | 61.23 | 53.95 |
| Mean  (SD) | 50.28  (9.17) | 58.51  (12.83) | 50.53  (6.01) | 63.60  (7.06) | 55.95  (15.60) | | 54.38  (11.17) | 61.89  (2.09) | 61.68  (12.42) |

*ZrO_2_: Group 1, IM ZrO_2_. ^†^ZrO_2_-S: Group 2, IM ZrO_2_-S. ^‡^Ti-T: Group 3, Ti-turned.

**Table S2.** Raw data of bone-to-implant contact and bone area ratios at 28 days after bone healing

| Rabbit No. | BIC (%) | | | | | BA (%) | | | |
| --- | --- | --- | --- | --- | --- | --- | --- | --- | --- |
|  | ZrO_2_* | ZrO_2_-S^†^ | Ti-T^‡^ | Ti-SLA | ZrO_2_ | | ZrO_2_-S | Ti-T | Ti-SLA |
| 5 | 45.76 | 70.49 | 52.47 | 71.64 | 32.07 | | 66.14 | 49.89 | 65.16 |
| 6 | 67.21 | 34.24 | 42.73 | 61.09 | 78.57 | | 34.65 | 41.58 | 58.85 |
| 7 | 43.02 | 64.79 | 29.41 | 70.52 | 33.76 | | 75.96 | 64.00 | 56.00 |
| 8 | 32.42 | 62.13 | 36.04 | 64.8 | 56.84 | | 62.84 | 43.73 | 62.82 |
| Mean  (SD) | 47.10  (14.59) | 57.91  (16.16) | 40.16  (9.84) | 67.01  (4.96) | 50.31  (21.98) | | 59.89  (17.73) | 49.80  (10.10) | 60.71  (4.08) |

*ZrO_2_: Group 1, IM ZrO_2_. ^†^ZrO_2_-S: Group 2, IM ZrO_2_-S. ^‡^Ti-T: Group 3, Ti-turned.
